# A Demonstration of Nesting in Two Antarctic Icefish (Genus *Chionodraco*) Using a Fin Dimorphism Analysis and *Ex Situ* Videos

**DOI:** 10.1371/journal.pone.0090512

**Published:** 2014-03-05

**Authors:** Sara Ferrando, Laura Castellano, Lorenzo Gallus, Laura Ghigliotti, Maria Angela Masini, Eva Pisano, Marino Vacchi

**Affiliations:** 1 Department of Earth, Environmental and Life Science (DiSTAV), University of Genoa, Genoa, Italy; 2 Costa Edutainment Spa, Acquario di Genova, Genoa, Italy; 3 Institute for Environmental Protection and Research (ISPRA) c/o Institute of Marine Sciences (ISMAR), National Research Council, Genoa, Italy; University of Basel, Switzerland

## Abstract

Visual observations and videos of *Chionodraco hamatus* icefish at the “Acquario di Genova” and histological analyses of congeneric species *C. hamatus* and *C. rastrospinosus* adults sampled in the field provided new anatomical and behavioral information on the reproductive biology of these white blooded species that are endemic to the High-Antarctic region. During the reproductive season, mature males of both species, which are different from females and immature males, display fleshy, club-like knob modifications of their anal fin that consisted of a much thicker epithelium. Histology indicated that the knobs were without any specialized glandular or sensorial organization, thus suggesting a mechanical and/or ornamental role of the modified anal fin. In addition, the occurrence of necrotic regions at the base of the thickened epithelium and the detachment of the knobs in post-spawning *C. hamatus* males indicated the temporary nature of the knobs. The role of these structures was confirmed as mechanical and was clarified using visual observations and videos of the behavior of two *C. hamatus* during a reproductive event that occurred in an exhibit tank at the “Acquario di Genova”. The reproductive process included pre-spawning activity, preparation of the nest, egg guarding and successfully ended with egg hatching. When the spawning event approached, the male prepared the nest. The nest was constructed on an accurately selected bottom surface, which was flattened and maintained free from sand or debris by a combination of radial body movements and continuous anal fin sweeping, thus demonstrating the important mechanical/abrasive function of the anal fin knobs. The present data are the first records of active nesting in icefish and clarify the meaning of dimorphic temporary structures, whose function would have been difficult to obtain in the field.

## Introduction

In the Southern Ocean, the Permanent Ice or High-Antarctic Zone [1] [2] is among the most challenging global marine environment. Nevertheless, these icy waters are inhabited by a rich and diverse ichthyofauna, whose evolution has been driven by the dramatic paleoclimatic and paleogeographic changes that originated the Southern Ocean [3].

Among the monophyletic perciform suborder Notothenioidei, which presently dominates the fish fauna of the Antarctic shelf [3], the icefish family (Channichthyidae) has unique features with the most notable being the absence of hemoglobin [4]. Of the 15 species included in the family (17 including two species in the genera *Cryodraco* and *Channichthys*), two-thirds live in High-Antarctic waters and only *Champsocephalus esox* is observed on the southern Patagonian shelf and in the Strait of Magellan [5]. The evolutionary loss of hemoglobin, which distinguishes icefish from other Antarctic and non-Antarctic fish but is also relative to other vertebrates, has led to the substantial remodeling of the cardiovascular system to facilitate adequate tissue oxygenation and a large suite of compensative physiological adaptations (reviewed in [6]). Furthermore, a variety of ecologically significant evolutionary changes are recognizable in icefish, such as reduced skeletal ossification and fat depositions in the viscera, which allows these fish, plesiomorphically without swim bladders, to obtain static lift, which is functional for benthopelagic habits.

Because of the broad spectrum of biological and ecological characteristics, icefish have been extensively studied (reviewed in [7]). However, most of the available data concerns fish living in the most accessible areas of the Southern Ocean, particularly those species targeted by commercial fisheries. By contrast, many ecological and biological issues must be fully clarified for species distributed in High-Antarctic areas [7].

Regarding reproductive biology and specifically reproductive behavior, fragmented data have been recently accumulated. Laying eggs in nests and parental guarding appear to be common strategies in channichthyid species and in the five lineages included in the Antarctic notothenioid clade [8]. The first record of egg guarding behavior in icefish was reported for *Pagetopsis macropterus* in 1969 [9]. However, the recent and more adequate availability of technology for underwater videos has confirmed *P. macropterus* parental care behavior [10] and documented *in situ* egg guarding in two additional icefish: *Chaenocephalus aceratus* [11] and *Chaenodraco wilsoni* [12]. In all cases, the eggs were guarded by individuals recognized as males according to their fin features [13].

Sexual dimorphism has been observed in several icefish species. A significant difference between sexually mature males and females was observed for the height of the dorsal fin in *Champsocephalus gunnari*, *C. wilsoni* and species of the genus *Chionodraco* [7]. Dimorphism of the anal fin rays was reported in *Chionodraco hamatus* and *C. wilsoni* [14]. In sexually mature males (gonads in maturity stages III, III–IV, IV, V and VI) of the above-mentioned species, the rays of the anal fin are mace-shaped and white with a probable outgrowth of connective tissue [14]. However, no additional data are currently available regarding the structure and function of these sexually dimorphic structures.

A wide variety of sexually dimorphic tegumentary structures linked to reproductive habits have been described in fish, including breeding tubercles and contact organs [15]–[17], fin knobs [18]–[20] and anal glands [21]–[23]. Secondary functions related to reproductive strategies, such as pheromones or antimicrobial substance production through glandular organs in the anal fin, have been occasionally demonstrated for specialized fins in males [21]–[23]. Fleshy lateral extensions of the anal fin rays in males of some blennid species have been documented as rubbing devices, which could be potentially useful to clean eggs [18]. In darters, freshwater Perciformes of the family Percidae, fleshy knobs have been described on the distal margin of male fins during the reproductive season and different roles have been suggested for these structures. In some cases, an egg mimicking function has been proposed as the most likely explanation for male fin specialization as a strategy to attract females to the nest. Moreover, it has been suggested that the egg mimicking fleshy knobs located on the distal margin of the dorsal fin originated to protect eggs from fin ray punctures in species in which the eggs are attached to the ceiling of the nest [19]. In another darter species, the knobs function to clean nesting substrates and “nest-gripping” has been proposed [24], [25].

In this context, our present study focused on the structure and function of sexually dimorphic anal fins in two congeneric icefish species, *Chionodraco hamatus* and *C. rastrospinosus*, endemic to the High-Antarctic region.

Three species, *hamatus*, *myersi* and *rastrospinosus*, are presently included in the genus *Chionodraco*. The former two species have a circum-Antarctic distribution, whereas *C. rastrospinosus* is confined to the Southern Scotia Arc and Antarctic Peninsula [7]. The three icefish species are typically epibenthic with a wide bathymetric distribution and range from shallow waters up to 1000 m deep [5].

*C. hamatus* and *C. rastrospinosus,* herein studied, differ in a few morphological characteristics: the presence of a rostral spine on the tip of the snout in *C. rastrospinosus* and a different number of gillrakers [26]. However, a comprehensive analysis integrating mitochondrial DNA with morphological data has recognized the validity of these two phylogenetically related species [27].

*C. hamatus* and *C. rastrospinosus* are both late summer/early autumn spawning species [7]. Sexual dimorphism has been described in *Chionodraco* sp. [7]. *C. hamatus* mature males and females differ based on dorsal fin height and body color [28]. No data are currently available on sexual dimorphism in *C. rastrospinosus*.

In the present study, the histological analysis of adult specimens sampled in the field were coupled with *ex situ* observations performed on two *C. hamatus* individuals during a reproductive event as it occurred in an exhibit tank at the “Acquario di Genova”. The integration of structural data and videos provided the first documentation of active nesting in a *Chionodraco* species and established a specific function of dimorphic anal fins during pre-spawning activities.

## Materials and Methods

### Sampling

Five adult *C. hamatus* were collected by trammel nets during the XIII Italian Antarctic Expedition on February 1998 in Terra Nova Bay (Ross Sea). Whole fish were frozen and preserved at −20°C. The frozen gonads and anal fins were later removed and fixed in paraformaldehyde at room temperature, washed in phosphate buffered saline (pH 7.4 and 0.1 M) and stored in 70% ethanol.

Eighteen adult *C. rastrospinosus* were collected by bottom trawling during the ANT XXVIII/4 Expedition on-board the R/V Polarstern (March-April 2012) in the Elephant Island – South Shetland Islands areas. Fifteen adult *C. hamatus* were collected by trammel nets in the Terra Nova Bay (Ross Sea) at the beginning of January 2013 during the XXVIII Italian Antarctic Expedition.

The *C. hamatus* sampling using trammel nets in the 1997–98 and 2012–13 Antarctic Italian Expeditions was authorized by the Italian National Antarctic Research Programme (PNRA) on behalf of the Italian Ministry of Foreign Affairs. The sampling was conducted in compliance with the “Protocol on Environmental Protection to the Antarctic Treaty”, Annex II, Art. 3, to provide specimens for scientific activity, referring to the PNRA Research Project. The specimens that remained alive after the capture were killed with a lethal dose of anesthetic MS 222 according to the European Commission Directive 86/609/EEC of November 24, 1986.

The *C. rastrospinosus* specimens were dead in the catches obtained by bottom trawl nets during the German Polarstern Research Cruise XXVIII/4 authorized by the CCAMLR (Commission for the Conservation of Antarctic Marine Living Resources) in accordance with Conservation measure 24-01 for scientific research activities in the CCAMLR statistical sub-area 48.1.

The gonads and anal fins from each specimen were fixed in paraformaldehyde, washed with seawater and stored in 70% ethanol.

All the specimens analyzed in present study were measured and weighed (see supplementary Table S1). The maturity of the gonads was evaluated by a macroscopic analysis according to the Kock and Kellermann five-point maturity scale [29].

### Histology

Samples of the anal fins and gonads were embedded in Paraplast (McCormick Scientific, USA) and 5-µm-thick sectioned. Hematoxylin–eosin staining (Bio-Optica, Milano Italy) was performed. The sections were examined using a Leica DMRB light microscope. The images were acquired using a Leica CCD camera DFC420C (Leica, Switzerland).

### *Ex situ* Observations

Some *C. hamatus* specimens were collected in the Terra Nova Bay (Ross Sea) in February 2004 and successfully transported to Italy on-board the M/N Italica for 49 days to place in a special Antarctic sector in the “Acquario di Genova” for educational purposes.

The living *C. hamatus* specimens transported to Italy were caught with permit issued by PNRA, Italy, on behalf of the Italian Ministry of Foreign Affairs and complied with the “Protocol on Environmental Protection to the Antarctic Treaty”, Annex II, Art. 3. No scientific or laboratory experiments were planned and fish maintenance was performed following the specific EU Council Directive 1999/22/EC of March 29, 1999 on the maintenance of wild animals in zoos, the related Italian legislation DL 21/03/2005, n. 73 and the EAZA (European Association of Zoos and Aquaria) Code of Practice. Therefore, approval by the IACUC or the equivalent animal ethic committee was not required.

The transport was performed in a special cold-conditioned container that was constantly maintained at −1°C and included 2 Life support systems, one for fish and another for invertebrates and a 1000-l reserve tank. The fish system consisted of 2 fiberglass tanks (approximately 800 l each), an automatically starting reserve pump with a magnetic drive, a mechanical sand filter and a biological filter with bio-balls. The fish were gradually introduced into the tanks (the specimens were assigned to a tank according to size) and fasted 3–4 days before the beginning of the journey to avoid overloading the water treatment system. The animals were fed with krill, which was cut into pieces if necessary based on the size of the receiving fish. Various techniques, including the use of nylon thread or wooden sticks, were used to overcome the reluctance of some fish to eat. The icefish showed the greatest reluctance to eat and were only successfully fed with live food such as live shrimp and small *Trematomus bernacchii*. Details on the life support system involved in the successful transportation of the icefish from Antarctica to the Acquario di Genova are reported in the “Rapporto sulla Campagna Antartica Estate Australe 2003–2004” (http://www.pnra.it/biblioteca/docs/rapporti_campagna/CA03-04.pdf). Further details will be widely provided in a specific technical publication. After acclimatization in insulated tanks placed in a cold room, a couple of icefish specimens were hosted in an exhibit tank (1 m^3^ volume and 1.14 m^2^ bottom surface covered by coarse gravel, sand and irregular rocks collected in the sampling area). The tank was a component of an integrated system of 5 tanks of different sizes and volumes. All tanks hosted Antarctic fish and invertebrates with a total volume of 2.22 m^3^.

The tank illumination varied according to season to mimic natural Antarctic conditions: light from 6 am to 9 pm in the winter (from December 21 to March 21) and from 10 am to 5 pm in the summer (from June 21 to September 21). One hour was either added or subtracted every 15 days during the remaining periods of the year.

The animals were routinely fed frozen capelin (*Mallotus villosus*) once a week. The theoretical feeding rate was 5% body weight/week.

An operator followed the reproduction events through daily observations from January 31 to July 31, 2007. The most significant reproductive steps were recorded using a digital video camera (Sony DCR PC110E). The specimens in the “Acquario di Genova” were maintained according to the current national and EU animal welfare regulations.

## Results

### Anal Fin Morphology

**Figure 1:**
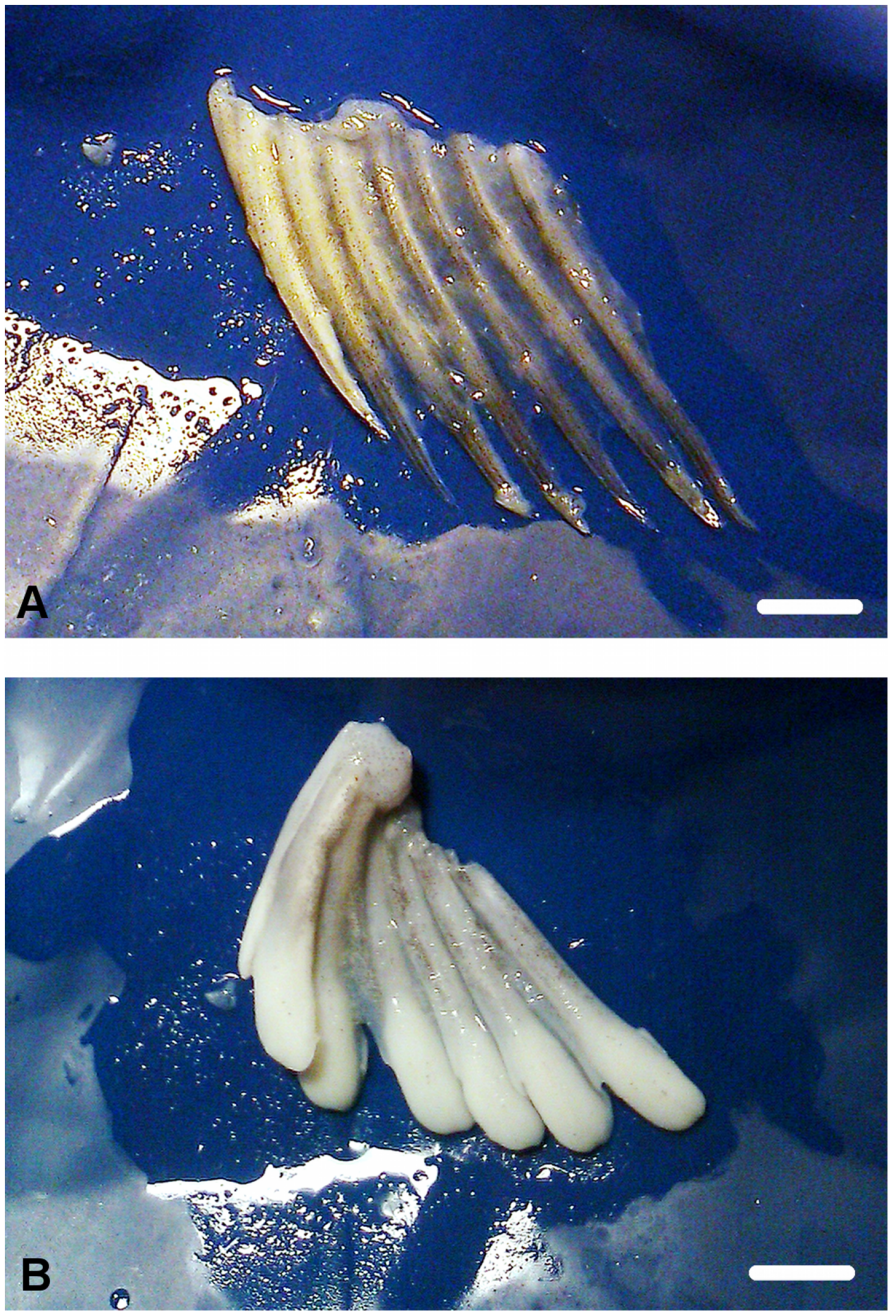
Anal fin rays of *C. hamatus*. (A) Immature male. (B) Mature male. Scale bars: 5 mm.

A macroscopic analysis of the anal fin in *C. hamatus* and *C. rastrospinosus* specimens emphasized the presence of two morphological variants relative to the appearance of the distal end of the fin rays: a) they were thin, sharp and light gray or b) thick, rounded and white (Figures 1A, B).

The measurements (antero-posterior) of the distal portion of an anal fin ray resulted in 1.5 mm (Figure 1A) for the a variant and up to 3–3.5 mm (Figure 1B) for the b variant.

### The Maturity Stage and Fin Knob Occurrence

**Figure 2:**
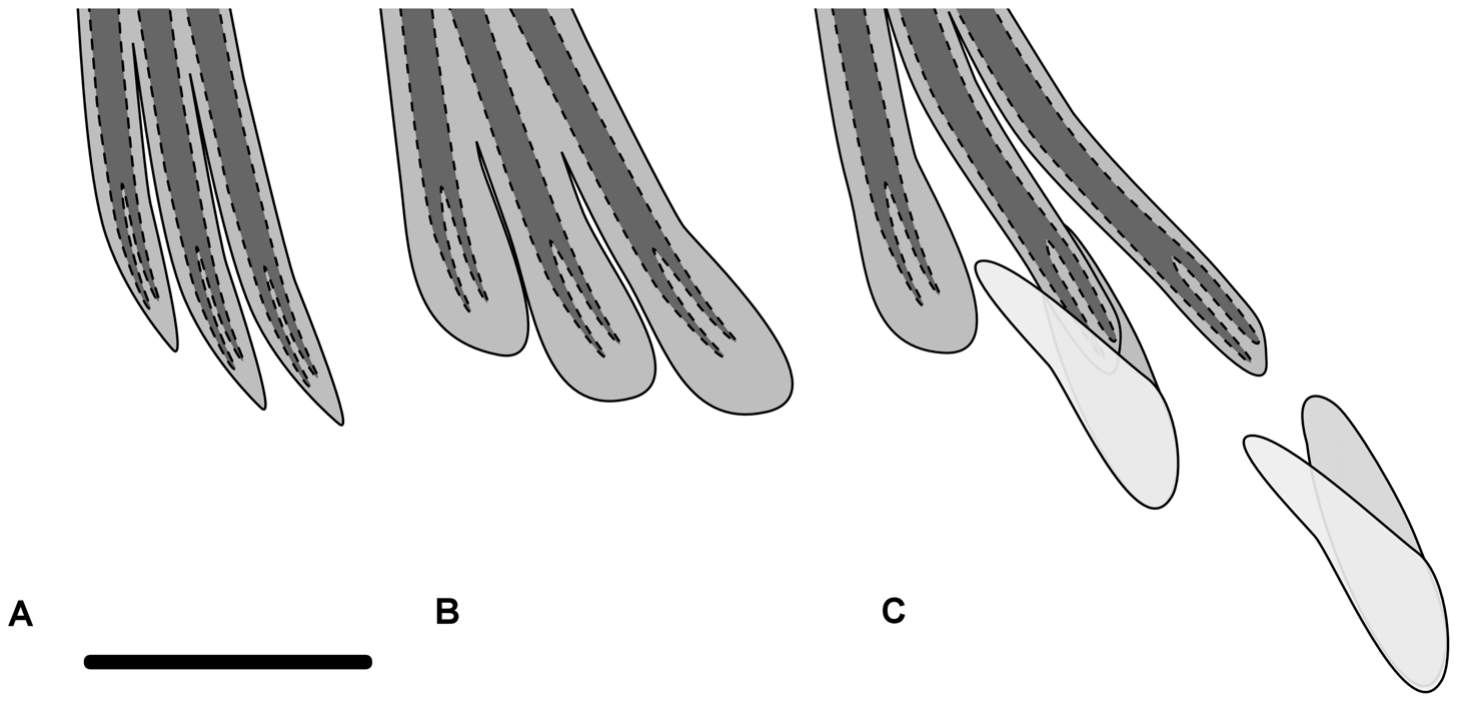
A diagram of an anal fin knob in *Chionodraco sp*. (A) The structure of anal fin rays in an immature male or female. (B) The structure of anal fin rays in a mature male. (C) The structure of anal fin rays in a post-spawning male. The distal enlargement detached easily from the fin. Dark grey = bony lepidotrichia, light grey = soft tissues. Scale bar: 10 mm.

In both species, females and immature males had thin and sharp fin rays (Figures 1A, 2A). Mature males had fleshy knobs on the anal fin (Figures 1B, 2B). Fin knobs have never been observed in females. In *C. hamatus*, detaching knobs or no knob have been observed in post-spawning male specimens (Figure 2C). The maturity stage evaluations of each specimen and data on the occurrence/absence of fin knobs are summarized in Table 1.

**Table 1:** Summary of the sampling, maturity stage and fin knob occurrence for each studied specimen.

Specimen	Date	Area	Sex	Maturity stage (macroscopic)	Gonadal stage (histology)	Fin knob	MNA Code
Chha1	4Feb998	TNB	M	4	Post-spawning	NO	MNA 5322
Chha2	4Feb998	TNB	M	4	Post-spawning	YES (detaching)	MNA 5323
Chha3	4Feb998	TNB	M	4	Maturing	NO	MNA 5324
Chha4	4Feb998	TNB	M	4	Post-spawning	YES (detaching)	MNA 5325
Chha5	4Feb998	TNB	M	4	Post-spawning	YES (detaching)	MNA 5326
Chha6	1Jan013	TNB	F	2	Pre-vitellogenic	NO	MNA 5327
Chha7	2Jan013	TNB	F	3	–	NO	–
Chha8	2Jan013	TNB	F	2	–	NO	–
Chha9	2Jan013	TNB	F	2–3	–	NO	–
Chha10	10Jan013	TNB	F	4	–	NO	–
Chha11	10Jan013	TNB	M	4	–	YES	–
Chha12	10Jan013	TNB	F	2	–	NO	–
Chha13	10Jan013	TNB	M	3–4	Spawning	YES	MNA 5328
Chha14	10Jan013	TNB	M	4	–	YES	–
Chha15	10Jan013	TNB	F	2	–	NO	–
Chha16	10Jan013	TNB	M	4	–	YES	–
Chha17	10Jan013	TNB	M	4	Spawning	YES	–
Chha18	10Jan013	TNB	M	4	Spawning	YES	–
Chha19	10Jan013	TNB	F	2	–	NO	–
Chha20	10Jan013	TNB	F	3–4	–	NO	–
Chra1	24Mar012	EI	M	1	Immature	NO	MNA 5329
Chra2	24Mar012	EI	M	3	Spawning	YES	MNA 5330
Chra3	24Mar012	EI	F	3–4	–	NO	–
Chra4	24Mar012	EI	F	3–4	–	NO	–
Chra5	24Mar012	EI	M	4	–	YES	–
Chra6	24Mar012	EI	F	4	–	NO	–
Chra7	27Mar012	SSI	M	3	–	YES	–
Chra8	27Mar012	SSI	F	3	–	NO	MNA 5331
Chra9	27Mar012	SSI	F	3	–	NO	–
Chra10	27Mar012	SSI	M	3	–	YES	–
Chra11	27Mar012	SSI	M	3	–	YES	–
Chra12	27Mar012	SSI	U	1–2	–	NO	–
Chra13	27Mar012	SSI	U	1	–	NO	–
Chra14	27Mar012	SSI	F	3	–	NO	–
Chra15	27Mar012	SSI	M	3	–	YES	–
Chra16	27Mar012	SSI	M	3	–	YES	–
Chra17	27Mar012	SSI	U	1	–	NO	–
Chra18	27Mar012	SSI	M	3	–	YES	–

Chha = *Chionodraco hamatus*; Chra = *Chionodraco rastrospinosus*; TNB = Terra Nova Bay; EI = Elephant Island; SSI = South Shetland Islands; MNA = The National Museum of Antarctica Felice Ippolito.

### Histology

**Figure 3:**
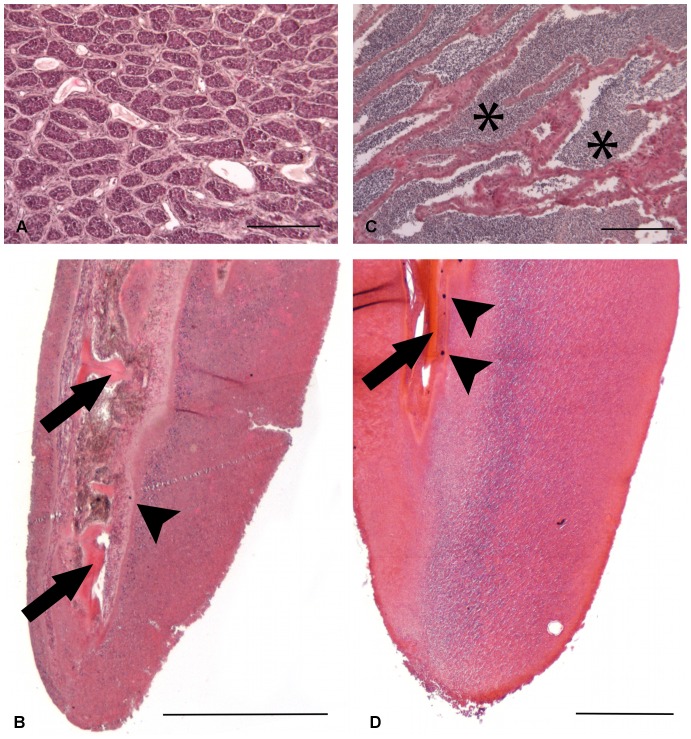
Histological sections of *C. rastrospinosus*, hematoxylin-eosin. A testis and a distal longitudinal section of an anal fin ray of an immature (A and B, respectively) and a mature (C and D, respectively) male. The immature male’s fin presents a thick epithelium and with this staining, only the fundamental cells of the epithelium are visible. Melanophores (arrowheads) and bony lepidotrichia (arrows) are recognizable in the connective tissue. In the mature male, mature spermatozoa are visible in the seminiferous tubules (asterisks). The distal end of the fin is covered by a thickened epithelium. Scale bars: (A) and (C) 100 µm; (B) and (D) 500 µm.

Immature males (see testes in Figure 3A) and females had fin rays without a knob and a tapered end (Figure 3B), whereas mature males (Figure 3C) had fin knobs covered with an epithelium, the thickness of which was variable according to the cutting plane but can reach 500 µm thick (Figure 3D). Some mucous cells were visible in the epithelium of the fin rays of all specimens, but none in the fin knobs. The connective tissue and bone elements were identically organized in all specimens: the bony lepidotrichia were branched in the distal portion (Figure 3B), whereas the melanophores were regularly disposed (Figure 3E).

**Figure 4:**
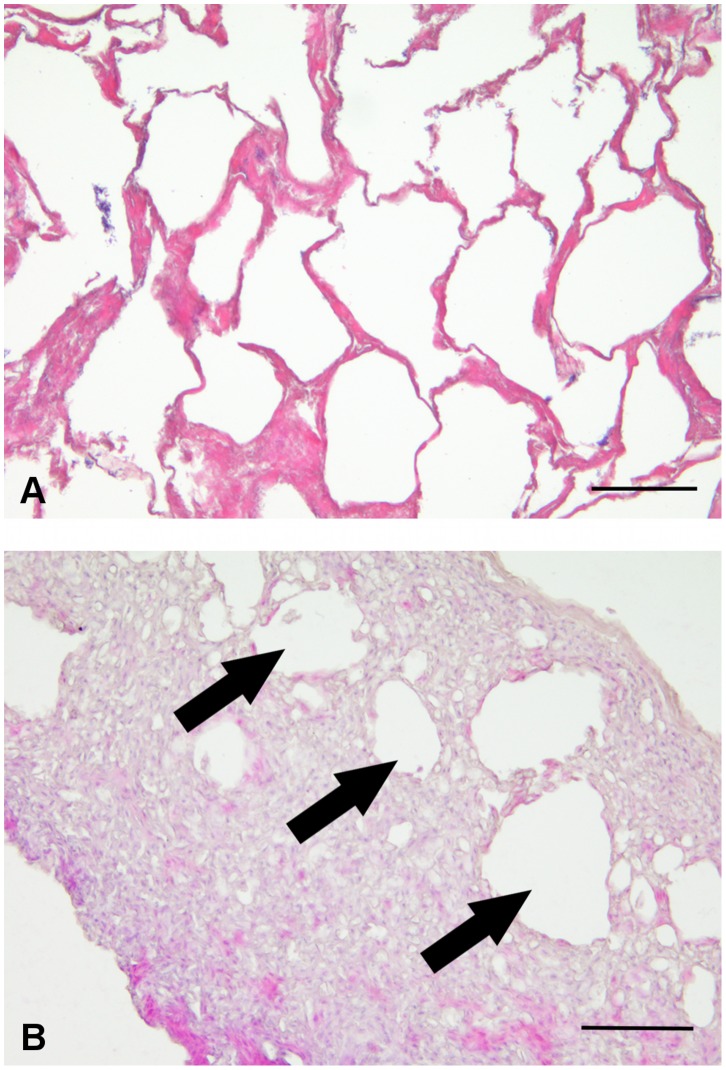
Histological sections of *C. hamatus*, hematoxylin-eosin. (A) A post-spawning testis. (B) A longitudinal section of a detaching anal fin knob with a wide, visible necrotic area (arrows). Scale bars: 100 µm.

The post-spawning (Figure 4A) *C. hamatus* knobs, which detached from the fin ray during sampling, showed tissue necrosis (Figure 4B).

### *Ex situ* Observations

After 3 years in an exhibit tank at the “Acquario di Genova”, a couple of *C. hamatus* reproduced. The reproductive process included pre-spawning activity, preparation of the nest and egg guarding and ended successfully with the hatching of viable, active hatchlings.

**Figure 5:**
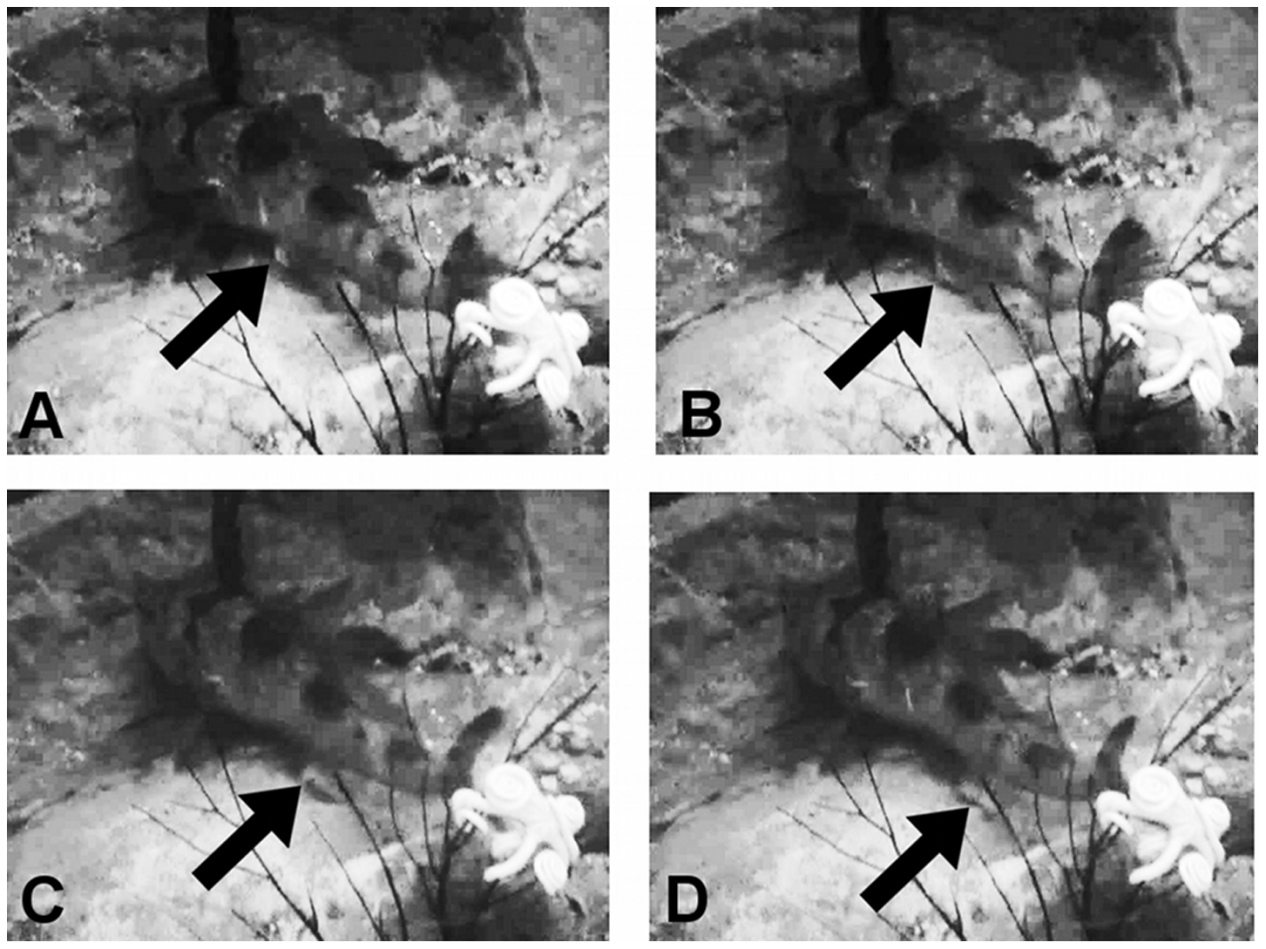
A sequence of still images of a *C. hamatus* captive male (Movie S1). (A-D) The wavy movement of the anal fin is emphasized by the arrow, which indicates the white knobs. Distance from each of these frames to the sequent: 0.08 sec.

The first recognizable evidence of spawning preparation began during the first week of February 2007. At that time, the female had a swollen abdomen and the male displayed anal fins with conspicuous knobs. In this phase, the male appeared to be the more active partner: he began moving around the tank to locate a potential nesting site and remained by this area. The male performed intense activities at the selected location as the spawning event approached: he leaned his pelvic fins against the bottom, performed regular, clockwise radial movements with his body and drew a circle on the substrate with a diameter that corresponded approximately with his body length. In addition, the male swept the substrate by vibrating the anal fin with an intense and continuous waving movement (Figures 5A–D; Movie S1). Resulting from the combination of body radial movements and fin sweeping, the surface chosen as the nest was abraded and flattened without sand and debris. The most intense substrate sweeping of the nesting area was performed on the day before spawning.

The male remained predominantly on the nest prior to spawning, whereas the female did not participate in the construction of the nest. In the pre-spawning period, the male infrequently moved close to the female to gently prod the swollen abdomen using his snout. The female responded to such behavior with rapid movements of her caudal fin. These interactions were infrequent and only lasted for a few minutes. The two fish did not eat prior to spawning.

The egg deposition, and presumably fertilization, occurred at night between February 8^th^ and 9^th^. The eggs were laid in the nesting site and scattered on peripheral areas where irregular rocky substrates occurred. From the first day after the eggs were laid, the female remained on the nest and typically leaned on her pelvic fins and moved her pectoral fins over the eggs in a fanning movement. We did not observe any direct egg care by the male during the embryo development period until hatching. The initial hatching occurred on June 26^th^: active hatchlings emerged from the eggs and rapidly swam toward the surface. Several other embryos were prepared to emerge from the chorion and hatching occurred progressively during July. The female continued egg guarding and fanning, alternating from continuous movement to interval fanning. The post-fertilization care continued even when all eggs were hatched or removed by the aquarium operators in an attempt to rear them in a more suitable nursery. On July 30^th^, the female ceased parental care and moved away from the empty nest. Egg size and quantity were consistent with species reports [30] [31] and will be detailed in a dedicated paper (Vacchi et al., unpublished data).

## Discussion

### The Function of Sexually Dimorphic Anal Fins in Chionodraco sp

Sexual dimorphism has been observed in a number of Channichthyidae species and is often related to the length of the first dorsal fin but also occasionally with other body measurements [7]. The presence of sex-linked anal fin knobs in *C. hamatus* was initially described by Gerasimchook [14]. The present study confirmed the result of Gerasimchook and also revealed similar sexually dimorphic anal fins in the co-generic species *C. rastrospinosus*.

According to present analysis of *C. hamatus* and *C. rastrospinosus*, there was a correlation between the rounded white anal fin knobs and mature males. The histological analyses excluded the presence of any sensory or glandular structures and assessed whether the fin knobs were comprised of a thicker epithelium. Moreover, the temporary nature of such fleshy knobs was shown, at least for *C. hamatus*, because they were observed to detach in the post-spawning stage with evident necrosis. The exclusive presence of fin knobs in mature males and their transient nature appears to imply the involvement of fin modification in reproduction. However, such a hypothesis would be difficult to demonstrate based only on morphological data.

The video presented herein provides evidence for the function of males’ modified anal fins in nesting. In the movie, the fish was obviously actively sweeping the sandy bottom with a combination of radial body movements and vibrations of the anal fin. A clean area was prepared for the eggs as a result of this well-combined, elegant and intense waving movements.

We cannot exclude that in addition to their evident role in nesting, the fin knobs might also play a role in the reproductive process of *Chionodraco* sp. as a secondary sexually dimorphic characteristic. In many species, dimorphic characteristics are involved in sexual selection and male-male competition. In many fish, females apparently prefer to spawn in nests that contain eggs and are attracted to males guarding eggs [32]. Egg-guarding males in some species evolved deception tactics based on egg-mimicking body ornaments, including egg-mimicking fleshy knobs, designed to attract females to prepared nests [33]. In the case of *Chionodraco* sp., the appearance of white and round anal fin knobs might indicate that a sexual selection strategy based on egg mimicry would apply. However, according to our observations at the “Acquario di Genova”, only the female *C. hamatus* guarded the eggs; therefore, the putative attraction effect of egg-mimicking fin knobs appears unlikely in this species. However, we are aware that captivity could influence reproductive behavior in some cases and further *in situ* data are necessary to corroborate this hypothesis.

In addition to a possible involvement in intersexual selection, the modified anal fin traits in *Chionodraco* males could function in an intra-sexual context as signals of maturity and strength. In some cichlid fish, a peculiar color pattern on the male anal fin, with a role in spawning behavior, have been experimentally related to differences in male aggressive behavior and not in female preference [34]. Therefore, although our observations were limited to two fish, we cannot exclude that conspicuous and evident fin knobs could allow *Chionodraco* males to gain deference from other males in a natural context and thus increase their reproductive success.

### Parental Care in Icefish

Teleostean fish display a remarkable variety of parental care strategies, which entail various forms of parental investment [35] and whose functional or adaptive roles are noted in the literature [36]–[39]. Nesting and egg guarding appear the most common behavior with males being the guarding sex more frequently than females [40]. According to the “association hypothesis”, female egg guarding often occurs in species with internal fertilization [41] [42]. By contrast, fish species characterized by external fertilization uncommonly present female egg guarding. In most cases, post-fertilization maternal care is applied for a short period (less than 1 day), as reported for the triggerfish *Rhinecanthus aculeatus* [43].

The parental presence on the nest presumably inhibits predation and decreases the amount of debris on the eggs, thus providing them with oxygen and assisting in the removal of waste [44] [45]. The benefits of nesting and parental care appear to extend beyond only increased survivorship of the offspring because an efficient nest maker and care offering parent influences mating success and partner choice. Nevertheless, parental care also has potential trade-offs: the guarding fish have fewer opportunities to feed while being exposed to higher predation risks [45]. Overall, parental care strategies certainly represent important species traits.

Nesting and egg guarding are also widespread among Antarctic notothenioid fish. In this suborder of fish, whose eggs are mainly demersal, egg- or nest-guarding behavior has been observed in all five notothenioid lineages of the Antarctic clade, including Channichthyidae (reviewed in Jones and Near [8]). Egg guarding was initially observed for *Harpagifer bispinis* in laboratory tanks and through *in situ* observations near the Antarctic Peninsula [46]. Subsequently, egg guarding has been observed in species from the family Nototheniidae, *Trematomus bernacchii* [47] and *Lepidonotothen nudifrons* [48]. Within Bathydraconidae, *Gymnodraco acuticeps* [49] and *Parachaenichthys charcoti* [50] have been observed by divers to guard eggs. Recently, egg guarding has been documented in the Artedidraconid species *Pogonophryne scotti* by *in situ* photographs [8]. Among icefish, *C. wilsoni*, *P. macropterus* and *C. aceratus* have been reported to deposit eggs on the seafloor or occasionally on the surface of flattened stones [7] [11] [12] [51]. In *C. wilsoni*, batches of eggs were observed with an ROV in adherent monolayers guarded by males [51]. *C. aceratus* males are described as tenaciously in contact with egg aggregates laid in shallow depressions on the bottom without macrobenthic organisms possibly by actions of the fish itself [11]. In addition to prior studies, an ROV survey in the coastal Ross Sea near the Terra Nova Bay recorded *P. macropeterus* guarding nests constructed on flat-bottomed areas or rocks [10]. Direct *in situ* observations of High-Antarctic icefish species have been historically limited by the seasonal accessibility of most coastal High-Antarctic areas. However, the breeding of icefish species in captivity has been challenging because of their peculiar biologic/physiologic features, although it has been occasionally performed for short periods of time. The nesting mechanisms in icefish were unknown because of difficulties in acquiring both *in-* and *ex situ* information data.

The present data are the first video documentations of nest construction behavior of the High-Antarctic species *C. hamatus*. In the video, the fish performs regular radial movements on the substrate while sweeping the substrate with anal fin vibrations in intense and continuous waving movements. Because of such a combination of body movements and anal fin sweeping, the surface chosen as nest was abraded, cleaned and prepared for the eggs. From our data, any chemical or anti-microbial substrate modification cannot be excluded or shown. However, the fish actively manipulated the substrate to prepare the candidate nest surface.

Such a nest construction based on substrate manipulation might be necessary in bottom areas crowded by benthos and rich in sediments, such as the reproduction sites of *C. hamatus*, to allow egg deposition. Although hardly quantifiable, the cost of the preparation of a clean nest surface is most likely well offset by a nest surface suitable for egg deposition and spatially delimited for easy guarding by the caregiving parent during long periods of egg guarding (approximately 5 months).

The nest construction activity and fin knobs themselves could also act as lures to attract females and induce them to lay their eggs in the selected area [52]. This suggestion appears to be supported by the pre-spawning behavior of the captive male approaching the female and prodding her swollen abdomen with his snout.

Although nest construction has only been observed in *C. hamatus*, the close phylogenetic relationship between the two *Chionodraco* species [27], their similar ecological habits and the shared sexual anal fin dimorphism described in this paper for *C. hamatus* and *C. rastrospinosus*, make it reasonable to hypothesize an identical nesting behavior in *Chionodraco* sp. Moreover, our data also support the possibility that *C. aceratus* would actively construct its nest, as presumed by Detrich and colleagues [11] based on the features of the nest and surrounding area.

## Supporting Information

Table S1 **Measurement of the** ***Chionodraco*** **specimens.**
(DOCX)

Movie S1 **A video of a** ***C. hamatus*** **captive male in the “Acquario di Genova”.**
(WMV)
