# Supplementary material for: A Demonstration of Nesting in Two Antarctic Icefish (Genus Chionodraco) Using a Fin Dimorphism Analysis and Ex Situ Videos
Source: PLoS One. 2014 Mar 5;9(3):e90512. doi: 10.1371/journal.pone.0090512 (PMC3944016; doi:10.1371/journal.pone.0090512)
Supplement: Table S1 — Measurement of the Chionodraco specimens. (DOCX) [file pone.0090512.s001.docx]

Table S1 – Measurement of the *Chionodraco* specimens

| Specimen | sex | Mean Depth (m) | Total Length (mm) | | Total weight (g) | Gonad weight (g) |
| --- | --- | --- | --- | --- | --- | --- |
| Chha1 | M | 165 | | 352 | 304 | 5,9 |
| Chha2 | M | 165 | | 353 | 325 | 7,3 |
| Chha3 | M | 165 | | 348 | 316 | 7,5 |
| Chha4 | M | 165 | | 355 | 346 | 7,4 |
| Chha5 | M | 165 | | 353 | 316 | 7,4 |
| Chha6 | F | 90 | | 375 | 248 | 0,98 |
| Chha7 | F | 90 | | 395 | 455 | 62,5 |
| Chha8 | F | 90 | | 395 | 355 | 3,86 |
| Chha9 | F | 90 | | 380 | 293 | 3,19 |
| Chha10 | F | 135 | | 402 | 608 | 174,40 |
| Chha11 | M | 135 | | 350 | 340 | 7,2 |
| Chha12 | F | 135 | | 410 | 428 | 4,8 |
| Chha13 | M | 135 | | 315 | 226 | 3,5 |
| Chha14 | M | 135 | | 360 | 387 | 10,26 |
| Chha15 | F | 135 | | 370 | 321 | 5,03 |
| Chha16 | M | 135 | | 360 | 357 | 6,4 |
| Chha17 | M | 135 | | 340 | 254 | 2,4 |
| Chha18 | M | 135 | | 370 | 360 | 7,4 |
| Chha19 | F | 135 | | 380 | 387 | 3,7 |
| Chha20 | F | 135 | | 380 | 560 | 160 |
| Chra1 | M | 197 | | 375 | 480 | 0,2 |
| Chra2 | M | 197 | | 422 | 732 | 11,0 |
| Chra3 | F | 197 | | 463 | 883 | 230,7 |
| Chra4 | F | 197 | | 473 | 1154 | 302 |
| Chra5 | M | 197 | | 440 | 649 | 17,2 |
| Chra6 | F | 197 | | 440 | - | - |
| Chra7 | M | 428 | | 422 | 550 | 4,5 |
| Chra8 | F | 428 | | 428 | - | 121,9 |
| Chra9 | F | 428 | | 385 | - | 117 |
| Chra10 | M | 428 | | 415 | - | 7,7 |
| Chra11 | M | 428 | | 393 | - | 4,0 |
| Chra12 | U | 428 | | 338 | - | 0,73 |
| Chra13 | U | 428 | | 306 | - | 0,2 |
| Chra14 | F | 428 | | 425 | - | 153,6 |
| Chra15 | M | 428 | | 405 | - | 4,7 |
| Chra16 | M | 428 | | 400 | - | 5,0 |
| Chra17 | U | 428 | | 238 | - | >0,1 |
| Chra18 | M | 428 | | 413 | - | 5,8 |

Chha= *Chionodraco hamatus*; Chra= *Chionodraco rastrospinosus*
